# New Insights into Avian Aerodynamic Valving: CT-Based CFD Analysis of Bronchial Airflow in the Domestic Chicken

**DOI:** 10.3390/ani16203250

**Published:** 2026-10-16

**Authors:** Longhuan Du, Li Yang, Chenming Hu, Mohan Qiu, Zengrong Zhang, Xiaoyan Song, Jiangxian Wang, Xia Xiong, Han Peng, Jialei Chen, Shiliang Zhu, Chunlin Yu, Chaowu Yang

**Affiliations:** 1 Sichuan Animal Science Academy, Chengdu 610066, China; longhuan_du@163.com (L.D.);; 2 Animal Genetic Breeding and Reproduction Key Laboratory of Sichuan Province, Chengdu 610066, China

**Keywords:** avian, respiratory tract, aerodynamic valving, unidirectional flow, CFD

## Abstract

The avian respiratory system, characterized by its caudocranial unidirectional pulmonary airflow, has long fascinated scientists. To date, the mechanisms underlying the unidirectional airflow and the formation of aerodynamic valving, especially the expiratory valve, remain poorly understood. To provide new insights into pulmonary airflow characteristics and to deepen our understanding of the role of aerodynamic valving in generating unidirectional pulmonary airflow, the respiratory tract of a domestic chicken was reconstructed based on CT-scanned data. CFD simulations were performed under various conditions after validation of the numerical model. Flow parameters were analyzed qualitatively and quantitatively during inspiration and expiration phases, respectively. The results showed that the coupled interaction of the distinctive bronchial topology and air-sac pressure conditions preferentially routed inspired air toward the caudal-sac pathways during inspiration. During expiration, aerodynamic valving was primarily associated with a momentum barrier generated by the cranial-sac airstream, which suppressed retrograde caudal-sac airflow through the primary bronchus and redirected it toward the dorsobronchial outlets leading to the physiological paleopulmonic pathway. Moreover, the ‘segmentum accelerans’ structure facilitated the formation of aerodynamic valving, whereas its removal did not abolish major-bronchial flow rectification, although higher air-sac boundary pressures were required during expiration. Finally, fluid density substantially affected inspiratory aerodynamic valving, whereas its effect on the critical pressures associated with expiratory valving remained below 5%.

## Simple Summary

Birds have a highly specialized respiratory system in which airflow through the gas-exchanging lung is predominantly unidirectional. However, the fluid–mechanical mechanisms that control airflow routing, especially during exhalation, are still not fully understood. In this study, we reconstructed the three-dimensional respiratory tract of a domestic chicken from CT images and used computational fluid dynamics to investigate airflow within the major bronchial system. The results showed that aerodynamic valving arises from the combined effects of airway geometry, pressure differences, and airflow inertia rather than from a discrete physical valve. During expiration, airflow associated with the cranial air sacs formed a momentum barrier that suppressed backward flow from the caudal air sacs through the primary bronchus and redirected it toward the dorsobronchial pathways. A narrowed airway region known as the ‘segmentum accelerans’ facilitated aerodynamic valving but was not essential for major-bronchial flow rectification. Fluid density also strongly influenced inspiratory aerodynamic valving. These findings provide new insight into the fluid mechanics of airflow regulation in the avian respiratory system.

## 1. Introduction

The avian respiratory system is a highly specialized respiratory design that differs markedly from the mammalian respiratory system [1], and has long captivated biologists for two primary reasons: First, its unidirectional pulmonary airflow provides a distinctive pattern of ventilation through the gas-exchanging lung. Second, the avian respiratory system, featuring avascular air sacs and highly vascularized lungs with complex airflow patterns, includes an air exchange structure (parabronchi) and a pump (air sacs) [2,3]. Importantly, unidirectional pulmonary airflow is not unique to birds. Farmer and Sanders [4] demonstrated bird-like unidirectional airflow in the lungs of the American alligator, indicating that this airflow pattern extends beyond Aves. Although decades of research have proposed a range of potential initial selective pressures for the evolution of unidirectional pulmonary airflow, such as heat conservation, water conservation, reduced cost of breathing, and improved crypsis, the formation and fluid dynamics mechanisms of the unidirectional airflow in avian lungs remain elusive to this day, as reviewed by Schachner and Moore in recent research [5].

### 1.1. Unidirectional Pulmonary Airflow: An Overview

The respiratory system of a typical bird, such as the domestic chicken, operates through a four-phase mechanism coordinated by air sacs, ensuring unidirectional pulmonary airflow.

**Figure 1:**
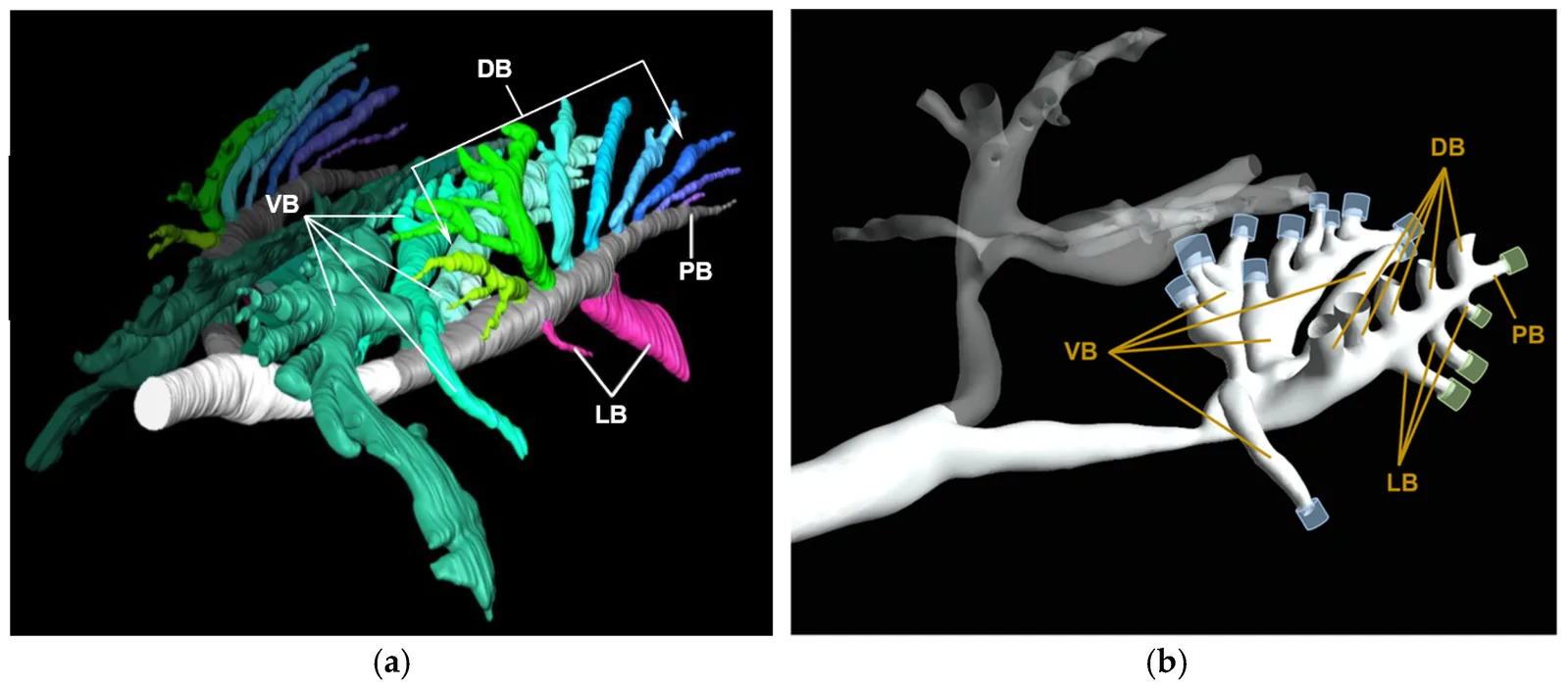
Solid representation of the bronchial tree of (**a**) the common ostrich (*S. camelus*), modified from Schachner et al. [10] (reprinted with permission from John Wiley and Sons) and (**b**) the common domestic chicken, from the present study. In image (**b**), the colored semi-transparent nubs indicate truncated bronchial surfaces used as surrogate CFD boundaries for air-sac-associated airflow pathways and should not be interpreted as the anatomical ostia of individual air sacs. The green nubs represent surrogate boundaries associated with caudal-air-sac pathways, whereas the blue nubs represent surrogate boundaries associated with cranial-air-sac pathways. The direct ostial connections of individual air sacs, including the proximal ventrobronchial connections, and the small paleopulmonic parabronchi connecting the DB and VB could not be reliably resolved from the present CT dataset and are therefore not explicitly represented.

**Phase 1—the first inspiration:** During inspiration, both the caudal and cranial air sacs are filled through different airflow pathways. Newly inspired air bypasses the ventrobronchi (VB, refer to Figure 1) and is directed either into the dorsobronchi or toward the caudal air sacs through the intrapulmonary primary bronchus and laterobronchi [5,6]. The negative pressure in caudal sacs, which facilitates this inspiration, is generated by the contraction of various muscles (e.g., the mm. intercostales externi, mm. levatores costarum, and the m. costosternalis [7,8]). Concurrently, air already present within the lung is drawn into the cranial air sacs.

**Phase 2—the first expiration:** Air from caudal sacs diverts into dorsobronchi (DB) rather than exiting through PB, subsequently entering paleopulmonic parabronchi and ventilating the lung caudally to cranially [9]. Expiration occurs due to the external pressure applied to the air sacs. Expiratory pressure arises from thoracic cavity reduction via contraction of the m. caudofemoralis and the mm. pubocaudales externus and internus combined with tissue elastic recoil [7,8].

**Phase 3—the second inspiration:** Concurrently with Phase 1, the air within the lung is drawn into the cranial sacs (including the cervical, cranial thoracic, clavicular air sac, etc.) by renewed negative pressure [9].

**Phase 4—the second expiration:** Cranial sac contents are expelled via PB to tracheal pathways, with Phase 4 synchronizing with Phase 2 during expiration.

Essential to this respiratory process, two complete cycles are necessary to achieve full air turnover. Consequently, the pulmonary tissues undergo continuous ventilation in a caudocranial trajectory, persisting through both the inhalation and exhalation [2].

### 1.2. The Aerodynamic Valving

Over the past several decades, researchers have been intrigued by the potential mechanisms underlying the unidirectional airflow in the avian lung. Early studies and speculations attributed this phenomenon to the presence of physical valves [11,12]. However, despite these hypotheses, no anatomical valves, such as sphincters or valve leaflets, have ever been identified in subsequent investigations [13,14]. Instead, a smooth-muscle constriction of the primary bronchus just proximal to the hilus of the lung, termed the ‘segmentum accelerans’, has been described in chickens (*G. gallus*), geese (*Anser anser*), and pigeons (*Columba livia*) [15,16]. Importantly, the ‘segmentum accelerans’ is not itself the inspiratory valve. Rather, inspiratory aerodynamic valving refers to the fluid-dynamic effect by which inspired airflow preferentially bypasses the ventrobronchial openings and is directed caudally, thereby limiting airflow into the ventrobronchi during inspiration [13]. Regarding the expiratory valve, a collection of physical models has shown that the angle and orientation of the airways play a crucial role in determining the direction and path of airflow [17]. Additionally, the speed of the airflow appears to be a significant factor in preserving the functionality of the ‘valve’ [18]. Nevertheless, due to the challenges associated with studying this issue from an anatomical perspective, the expiratory valve remains much less well understood compared to the inspiratory valve [5].

### 1.3. Aims of Present Study

With the advancement of computer technology, three-dimensional imaging techniques now enable the visualization of intricate structures within the avian lung that remain challenging to investigate in situ. Moreover, CFD simulations enable detailed visualization and quantitative analysis of airflow under prescribed operating conditions, complementing previous physical-model studies. Therefore, to provide new insights into the fluid mechanics of aerodynamic valving and strengthen our understanding of major-bronchial airflow routing in the avian respiratory system, this study employed computed tomography (CT) scanning, 3D printing, and computational fluid dynamics (CFD) technologies to:

- Construct a three-dimensional CFD model of the domestic chicken respiratory tract based on CT scanning data.
- Validate the computational model using a 1:1 scale 3D-printed physical model through experimental measurements.
- Simulate and visualize dynamic inspiratory airflow and steady-state expiratory airflow under prescribed boundary conditions.
- Qualitatively and quantitatively analyze inspiratory and expiratory aerodynamic valving and the associated airflow partitioning within the major bronchial system.

## 2. Materials and Methods

### 2.1. Model Reconstruction and CFD Setup

#### 2.1.1. CT-Based Respiratory Tract Reconstruction

**Figure 2:**
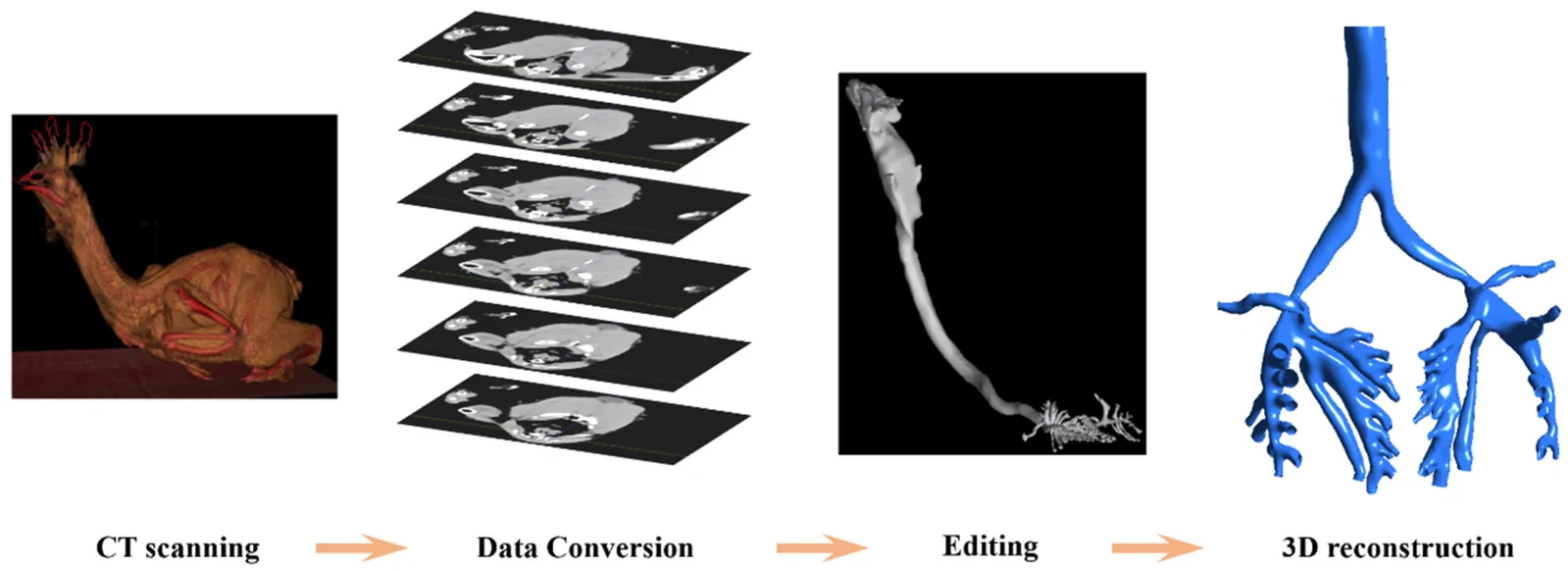
CT-based respiratory tract reconstruction workflow.

The anatomical structure of the respiratory system in a domestic chicken was reconstructed utilizing a computed tomography (CT) dataset derived from a healthy broiler weighing approximately 3.2 kg. This dataset encompassed the oral cavity, nasal cavity, oropharynx, larynx, trachea, and the tracheobronchial tree down to the third branching generation. Before initiating the CT scan, the chickens were euthanized and mechanically ventilated through a tracheal tube inserted via the mouth to fill its respiratory tract with air. Concurrently, to best replicate the respiratory tract structure of the chicken in its standing posture, plastic fixtures were used to clamp and secure the comb, ensuring that the neck was relatively maintained in a straight configuration as illustrated in Figure 2. The scanning procedure was conducted from the cranial to caudal end of the chicken using a Quantum 752 128-Slice scanner (Kuanteng, Beijing, China), configured with a 500 mm field of view, 70 kV, and 50 mA. The resulting CT dataset consisted of 836 sequential images, each with a slice thickness of 0.5 mm, and these images were captured in the Digital Imaging and Communications in Medicine (DICOM) format. Following this, the CT images were transferred to an image processing software (MIMICS 2024) for further refinement, including editing, smoothing, and three-dimensional reconstruction. Ultimately, the reconstructed geometry was transformed into the Standard Tessellation Language (STL) format to facilitate the subsequent mesh generation and 3D printing processes. A schematic diagram depicting the reconstruction workflow is presented in Figure 2. It should be noted that owing to the spatial resolution of the clinical CT dataset (slice thickness, 0.5 mm), the thin septa separating adjacent proximal ventrobronchial ostia could not be reliably resolved, and some adjacent branches appeared partially merged during segmentation. Consequently, the first ventrobronchus (V1) was not represented as a distinct airway in the reconstructed CFD geometry. The proximal ventrobronchial region should therefore be regarded as a simplified reconstruction rather than a complete representation of the native anatomy.

#### 2.1.2. CT-Based Geometry Modification

**Figure 3:**
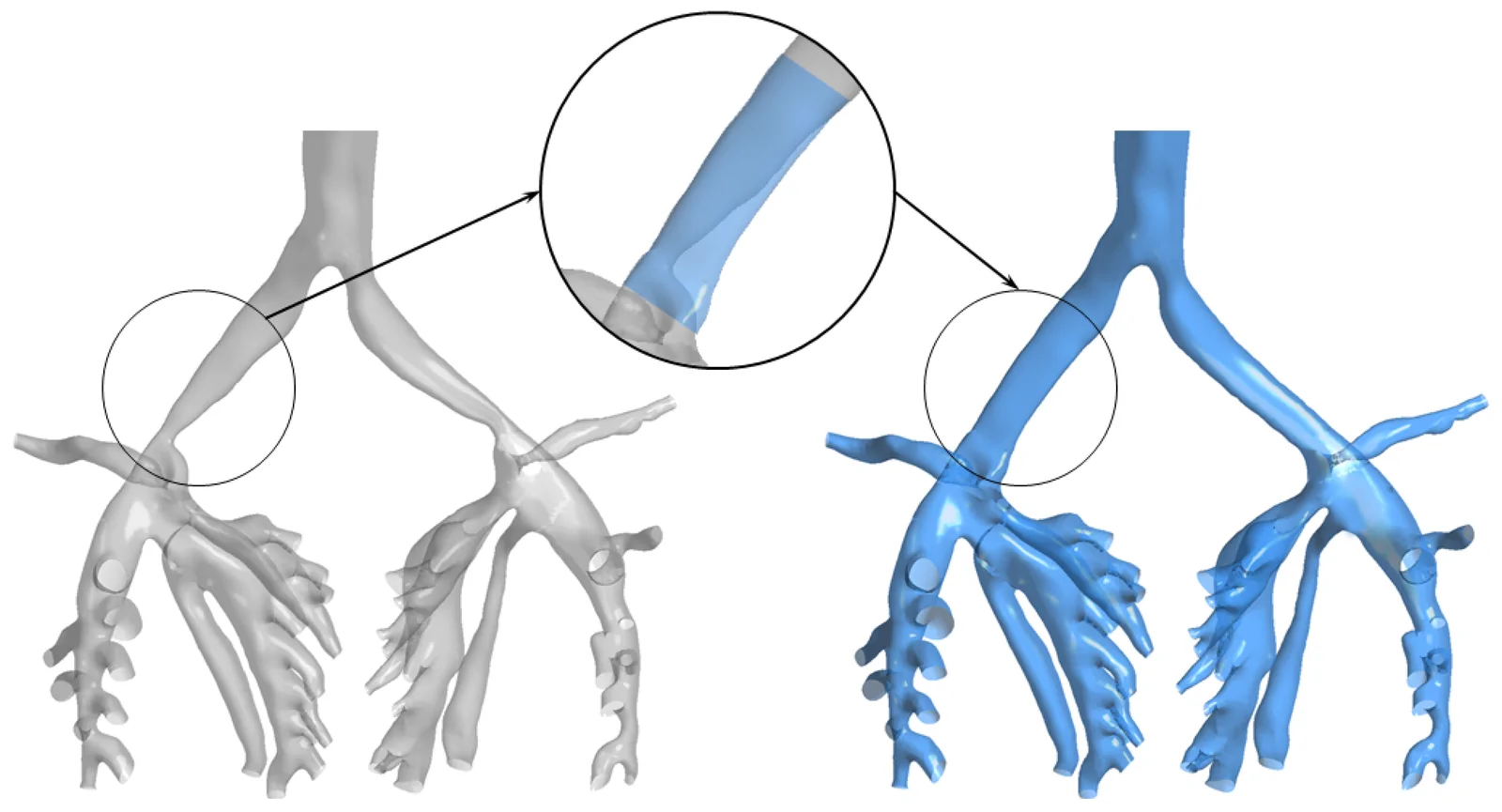
Schematic drawing of the CT-based modification of the ‘segmentum accelerans’ for comparative examination.

To provide a more comprehensive comparison of the effects of the ‘segmentum accelerans’ on airflow characteristics, flow partitioning, and aerodynamic valving within the major bronchial system, the ‘segmentum accelerans’ structure was artificially widened (see Figure 3) and simulated under identical boundary conditions, following a similar methodology to that employed by Maina et al. [19]. Notably, the modification was based on the subject-specific CT-derived geometry, allowing the effect of the ‘segmentum accelerans’ to be examined while preserving the remaining bronchial topology.

#### 2.1.3. Computational Mesh and Regional Division

**Figure 4:**
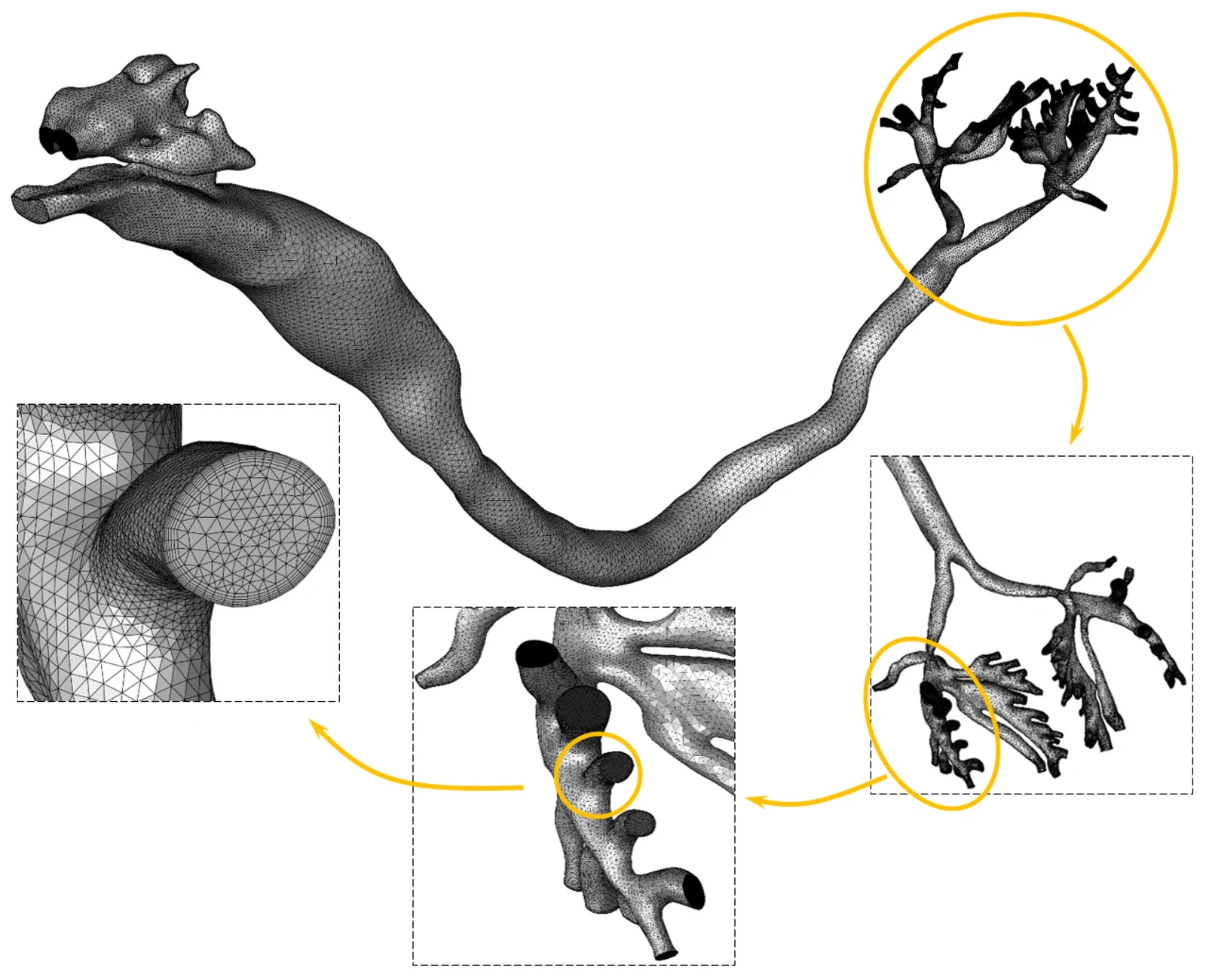
Numerical model mesh with detailed boundary treatment.

The numerical mesh for the respiratory tract of the chicken was created using ANSYS ICEM 2020 software. Initially, the mesh framework was established through the octree approach to generate a suitable surface mesh. Subsequently, a Delaunay triangulation-based technique was utilized to produce a high-quality volumetric mesh. Finally, six layers of prism mesh were applied near the airway walls to resolve near-wall velocity gradients, as shown in Figure 4. Particular emphasis was placed on the height of the first layer of the prism mesh to ensure that $y^{+}<1$ throughout the entire computational domain, which was recommended when employing the SST k−ω turbulent model.

**Figure 5:**
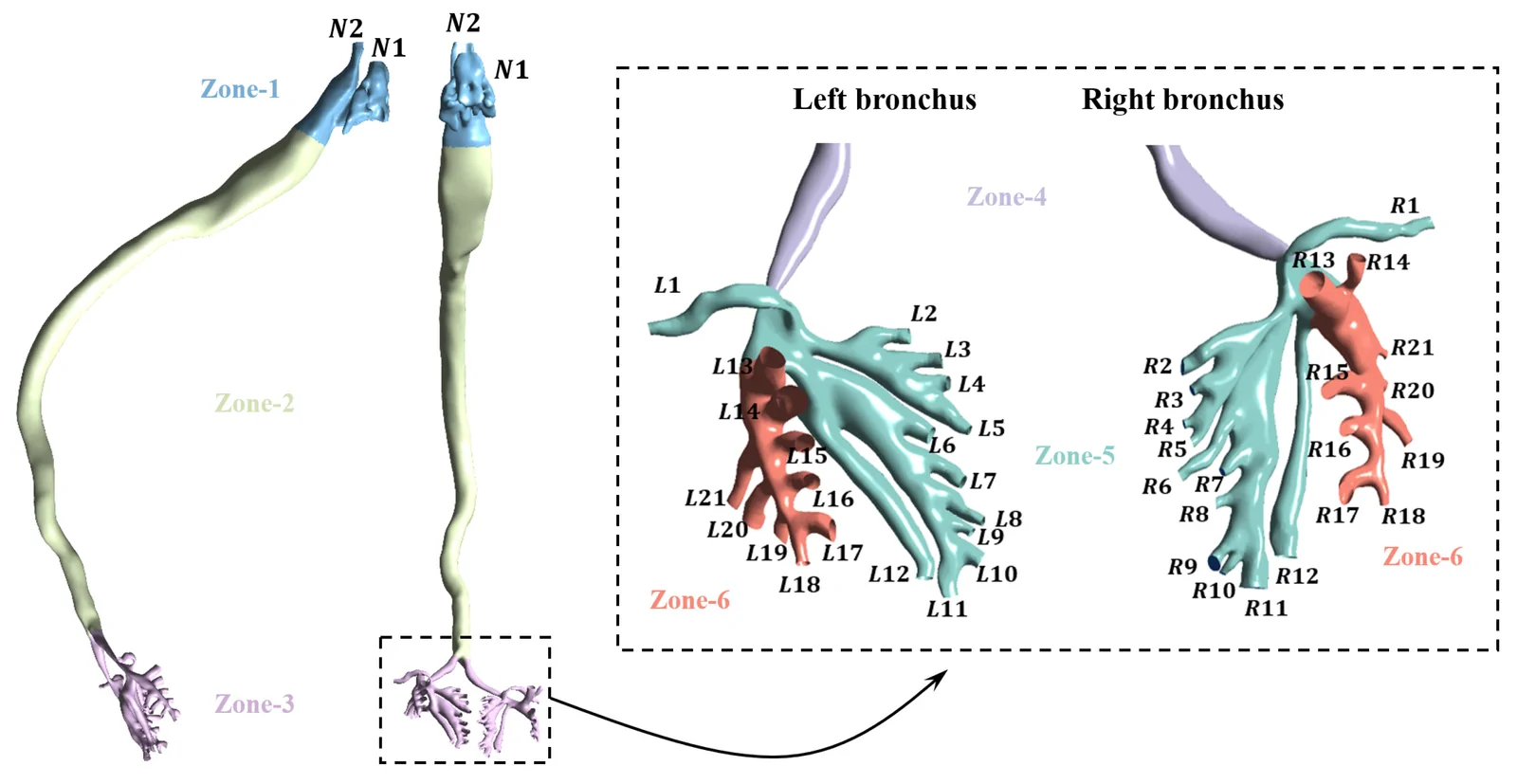
Different views of the entire airway and details of the regional division.

Moreover, to enable a thorough qualitative and quantitative investigation of the flow structure and field parameters within the entire respiratory tract, the full geometry was divided into distinct segments (see Figure 5): the Zone-1 region (including the oral cavity, the nasal cavity, the oropharynx and larynx), the Zone-2 region (the trachea) and the Zone-3 region (the lower respiratory tract), which could be further subdivided into Zone 4~6. Additionally, the nasal and oral inlets were designated as $N_{1}$ and $N_{2}$, respectively. Each left bronchial outlet was numbered with the letter ‘L’, while the right bronchial outlets were numbered with the letter ‘R’, thereby facilitating a detailed study of airflow direction during respiration.

#### 2.1.4. Boundary Condition

In this study, inhalation and exhalation were investigated separately. This approach was necessitated by the exclusion of the paleopulmonic parabronchi from the model, given that their minuscule size precludes segmentation from the CT dataset. As a result, it was not possible to directly simulate the complete respiratory process.

**Figure 6:**
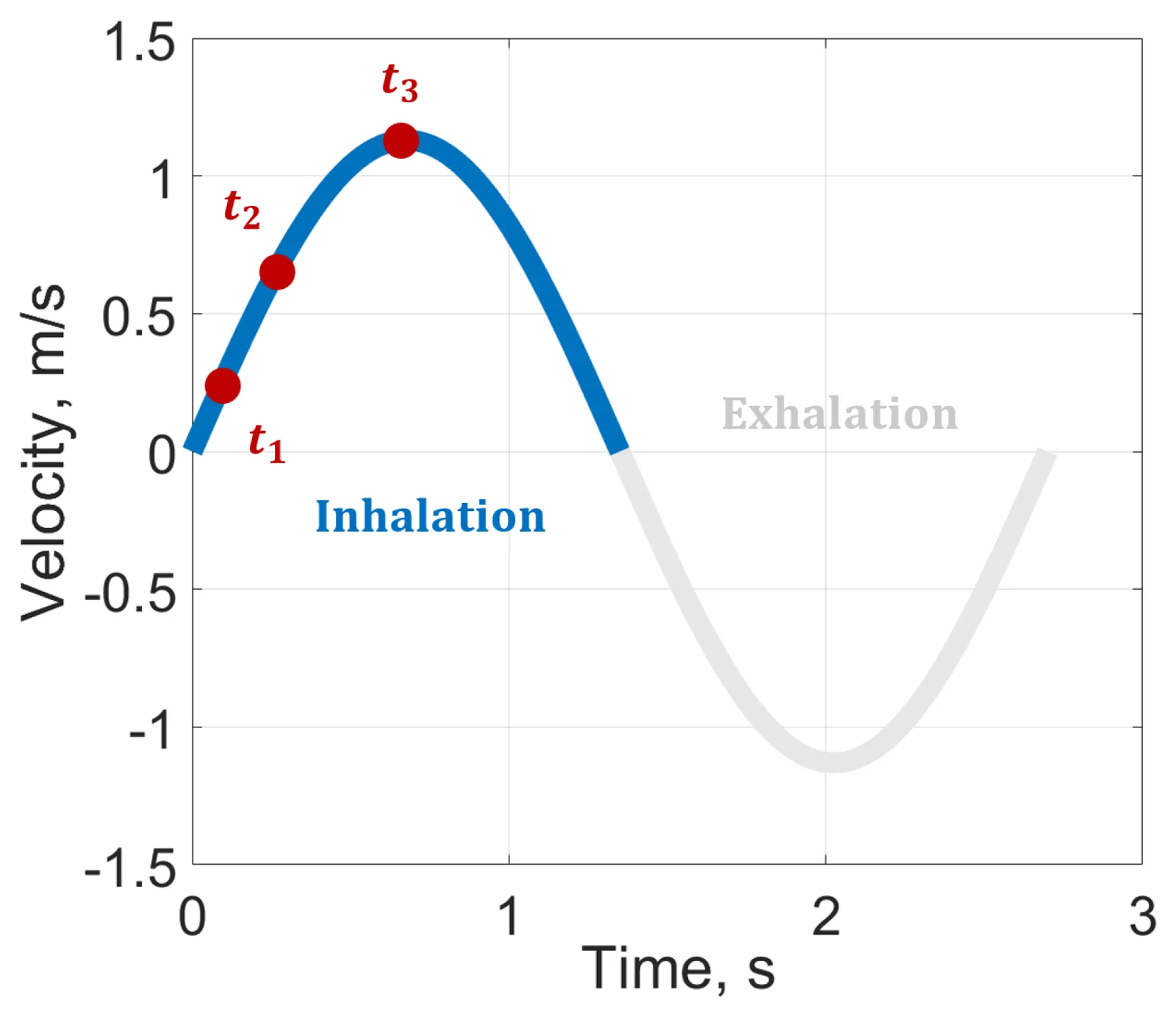
Simplified sinusoidal velocity inlet during inspiration phase. Investigations were performed at $t_{1}=T/20,t_{2}=T/8,t_{3}=T/4$

**Inhalation:** a velocity inlet was applied at the nose N1 (see Figure 5) of the animal to simulate its dynamic inspiration during the resting state. The airflow velocity fluctuated over time in accordance with the respiratory process and was modeled using a sinusoidal waveform as described in Equation (1) and illustrated in Figure 6:

(1) $$V=\frac{Q}{A}\cdot sin(\frac{2\pi }{T}\cdot t)$$

where Q was the flowrate in L/min, A was the cross area of the nose inlet in $m^{2}$, T was the duration of one complete respiratory cycle in seconds, s, and t was the simulation time step. Based on the study conducted by Nascimento et al. [20], the minute volume of a typical broiler chicken weighing 3.2 kg was estimated to be around $Q=1.7\;L/min(Q=2.83\times 10^{-5}\;m^{3}/s)$, and the corresponding tidal volume and respiratory rate were estimated to be approximately 77 mL/respiration and 22 respirations/minute, respectively. The cross-sectional area of the nose inlet was calculated to be approximately $A=2.5\times 10^{-5}m^{2}$.

All bronchi outlets L1−L21 and R1−R21 (see Figure 5) were set as pressure outlet boundary conditions of 0 Pa. Investigations about the flow parameters and characteristics of the inspiratory valve were performed at three time points ($t_{1}=T/20,t_{2}=T/8,t_{3}=T/4$) during the dynamic inspiratory phase (see Figure 6). Meanwhile, under the same simulation conditions, effects of the modification of the ‘segmentum accelerans’ were examined and compared.

**Exhalation:** Although current CT resolution did not allow reliable reconstruction of the small paleopulmonic parabronchi connecting the DB and VB, these pathways are present in the physiological avian lung. Therefore, the DB–parabronchi–VB pathway could not be explicitly represented in the present model. For the expiratory simulations, all segmented terminal VB branches (L1–L12 and R1–R12) were consequently treated as surrogate boundaries of the cranial air sacs and assigned the corresponding cranial-sac pressure boundary condition. This simplification enabled investigation of the interaction between the caudal- and cranial-sac airstreams, although the complete intrapulmonary flow pathway through the parabronchi was not resolved. Accordingly, a physiologically complete transient simulation of expiration was not feasible, and steady-state simulations under different pressure conditions were instead used to investigate the mechanisms governing major-bronchial expiratory aerodynamic valving.

For the purposes of the present model, an expiratory aerodynamic-valving condition was operationally defined as the state in which caudal-sac-derived airflow no longer propagated cranially through the primary bronchus toward the trachea and was instead redirected toward the DB outlets. Because the paleopulmonic parabronchi were not explicitly reconstructed, this criterion describes flow rectification at the level of the major bronchial system.

During the actual expiratory phase, external pressure (muscle contraction) was applied to both the caudal and cranial sacs. Air was forced from the caudal sacs into the lungs, while air in the cranial sacs was simultaneously expelled toward the upper airway. Due to the flow losses, the pressure at the DB boundary was assumed to be lower than that imposed at the caudal-sac boundaries (the LB and PB). For the simplified steady-state expiratory simulations, the boundary pressure was adjusted until the mass flowrate through the surrogate VB boundaries toward the upper respiratory tract matched that through the caudal-sac boundaries. Finally, it should be noted that the contraction and expansion of the air sacs were simulated by modifying the pressure boundary conditions of the corresponding bronchial outlets, with the air-sac ostia themselves not explicitly modeled. The boundary conditions for exhalation simulations were set as following steps:

1. The pressure at the DB outlets (corresponding to bronchi L13-L17 and R13-R17), representing the downstream pressure at the entrance to the unresolved paleopulmonic pathway, was preset based on the actual physiological pressure ranges reported in the literature [17,21,22], for example $P_{DB}$ = 1 Pa.
2. The pressure assigned to the caudal-sac boundaries (i.e., the LB and PB boundaries, corresponding to bronchi L18-L21 and R18-R21) was set to a value theoretically higher than that of the DB, for example $P_{LB}=P_{PB}$ = 1.2 Pa.
3. Set an initial pressure value for the VB boundaries, $P_{VB}=0$ (see Figure 5, bronchi L1–L12 and R1–R12), and run the simulation.
4. The mass flowrate through the caudal sacs (via LB and PB) was determined and recorded.
5. The pressure applied to the VB boundaries ($P_{VB}$) was then iteratively adjusted until the mass flowrate through these boundaries (VB) matched that through the caudal-sac boundaries. The corresponding VB boundary pressure was then recorded.
6. Once Step 5 was achieved, the airflow pattern was examined to determine whether the predefined expiratory aerodynamic-valving condition had been reached; namely, whether caudal-sac-derived airflow no longer propagated cranially through the primary bronchus toward the trachea and was instead redirected toward the DB outlets. If this condition was not achieved, the caudal-sac pressure was readjusted from Step 2, and Steps 2–5 were repeated (for example try $P_{LB}=P_{PB}$ = 1.5 Pa).
7. Through this iterative procedure, the critical boundary pressures required to achieve the modeled expiratory aerodynamic-valving condition were determined for each prescribed DB pressure. In this study, multiple DB pressures were tested, including $P_{DB}$ = 1, 3, 5, 10, 15, 20, 30, 40, and 50 Pa.

Furthermore, to investigate the effects of fluid density on airflow characteristics, major-bronchial flow partitioning, and aerodynamic valving, comparative simulations were conducted using air properties corresponding to sea level and an altitude of 10,000 m (the density is approximately one-third of the original). Finally, the nose and mouth were assigned distinct boundary conditions corresponding to the inhalation and exhalation phases, respectively. All boundary conditions are summarized in Table 1.

#### 2.1.5. Simulation Setup

ANSYS Fluent 2021 software was employed for all simulations. The Reynolds-averaged Navier–Stokes (RANS) equations were solved using the SIMPLEC algorithm to couple pressure and velocity. To enhance accuracy, the second-order upwind scheme was used for discretizing the transport equations. Drawing on similar studies in the literature (e.g., [23,24]), the shear stress transport (SST) k−ω turbulent model was selected. Although the Reynolds numbers were generally low, the complex airway geometry, including bends, constrictions, expansions, and multiple bronchial bifurcations, may induce locally disturbed, transitional, or even turbulent flow; similar behavior has been experimentally observed in respiratory airway models even at Re≈1500 [25]. The SST k−ω model was therefore adopted because of its suitability for low-Reynolds-number near-wall flow and its ability to predict adverse-pressure-gradient and separated flows. Convergence was achieved when all variables were below $10^{-5}$ between two consecutive iterations.

### 2.2. Grid Independency Study and Model Validation

#### 2.2.1. Grid Independency Examination

**Figure 7:**
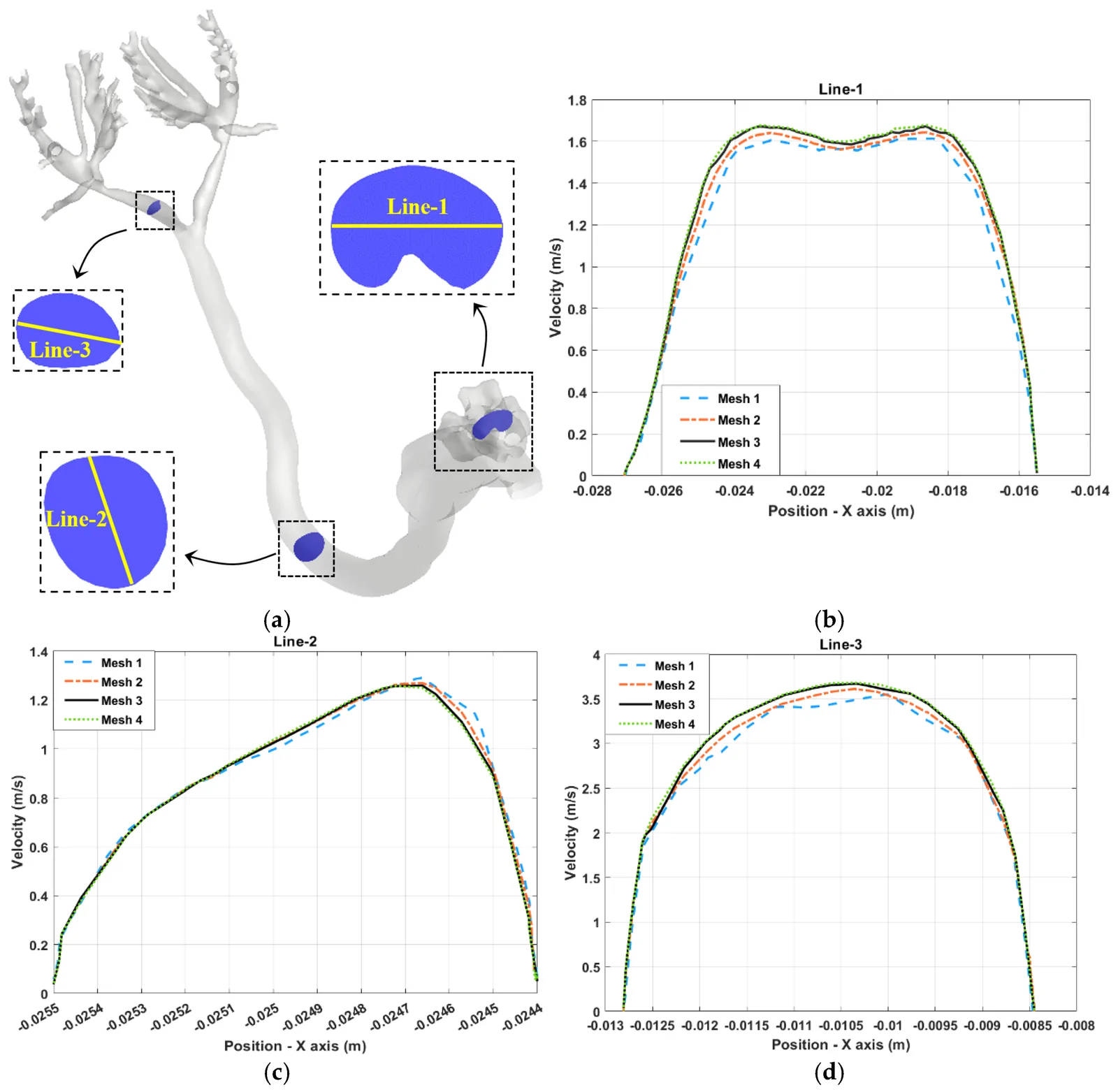
Mesh independency examination at the tidal volume of 60 mL/respiratory. (**a**) Schematically drawing of the locations of three cross-sections around the nasal cavity, the trachea and the bronchus, along with the definition of lines. (**b**) Velocity profiles at Line-1. (**c**) Velocity profiles at Line-2. (**d**) Velocity profiles at Line-3.

Given the highly complex geometry of the respiratory tract model, which included a multitude of anatomical intricacies, employing a sufficiently fine mesh was essential to accurately represent the geometry and produce reliable simulation outcomes. To this end, a grid independence test was conducted using four distinct meshes with different element cells: approximately 1.2 million (Mesh 1), 1.9 million (Mesh 2), 3.3 million (Mesh 3), and 4.5 million (Mesh 4). The axial flow velocities at three specific cross-sections—namely, the nasal cavity, the trachea, and the bronchus—were analyzed and plotted. This was done to assess the impact of varying mesh resolutions on the velocity profiles. As clearly demonstrated in Figure 7b–d, when the number of elements is increased from 3.3 million in mesh 3 to 4.5 million in mesh 4, the difference between the two meshes is minimal. The maximum velocity variation observed is merely 1.69%. Therefore, taking computational efficiency into account, mesh 3 was chosen for all subsequent simulations.

#### 2.2.2. CFD Model Validation

**Figure 8:**
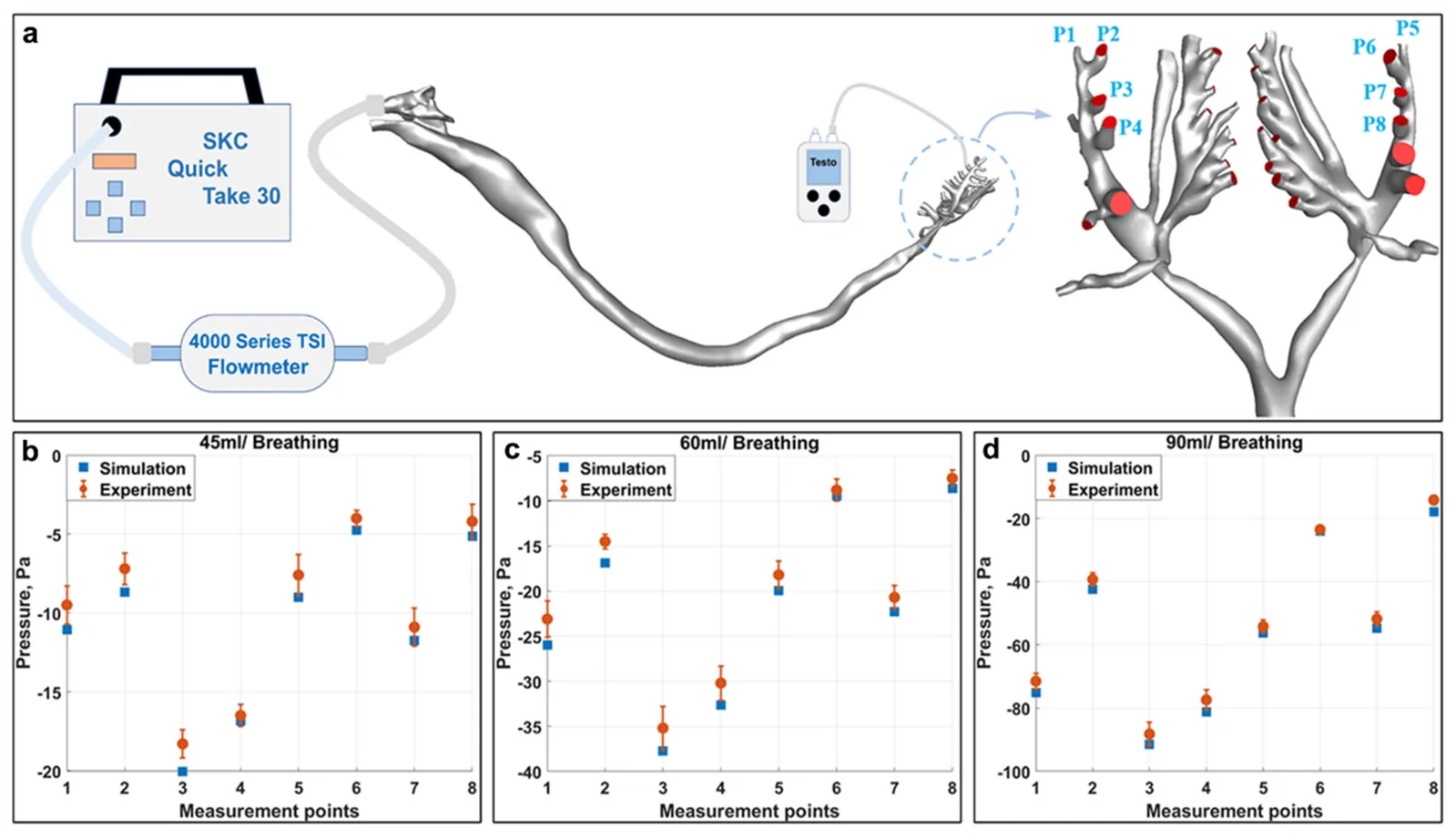
(**a**) Schematic drawing of the experimental configuration for pressure measurement at designated points P1 to P8. Comparison between the measured and simulated pressure values at the tidal volume of (**b**) 45 mL/Breathing, (**c**) 60 mL/Breathing, and (**d**) 90 mL/Breathing.

To verify the accuracy of the developed 3D CFD model, a full-scale, high-resolution physical replica of the respiratory tract was created using 3D printing techniques. The choice of material for this model was Acrylonitrile Butadiene Styrene (ABS), which was selected for its cost-effectiveness and sufficient durability. In the experimental setup, pumps fitted with flow meters were employed to deliver precise airflow rates, while a pressure gauge was used to record pressure readings at various locations of P1~P8, as illustrated in Figure 8. Detailed information regarding the experimental apparatus is summarized in Table 2. The validation process involved testing three tidal volumes (45 mL/Breathing, 60 mL/Breathing, and 90 mL/Breathing), which covered the range of respiratory volumes intended for subsequent simulations. As demonstrated in Figure 8b–d, the simulated values generally align well with the measured pressure data. It is suggested that the slightly higher pressures (low negative pressure values) observed during experiments may be attributed to the relatively high surface roughness of the unpolished internal surface of the physical model, which could lead to lower velocities. Conversely, this surface roughness effect was not considered in the numerical simulations. Meanwhile, the root mean square error (RMSE) corresponding to flowrates of 45 mL/Breathing, 60 mL/Breathing, and 90 mL/Breathing was found to be merely 1.2 Pa, 1.8 Pa, and 3.1 Pa, respectively. Therefore, despite the inherent complexity of turbulent flow dynamics and geometric intricacies in the airway model, the computational simulations produced satisfactory predictions [23,26], thereby confirming the validity of the developed numerical model.

## 3. Results

### 3.1. Inspiratory Phase

**Figure 9:**
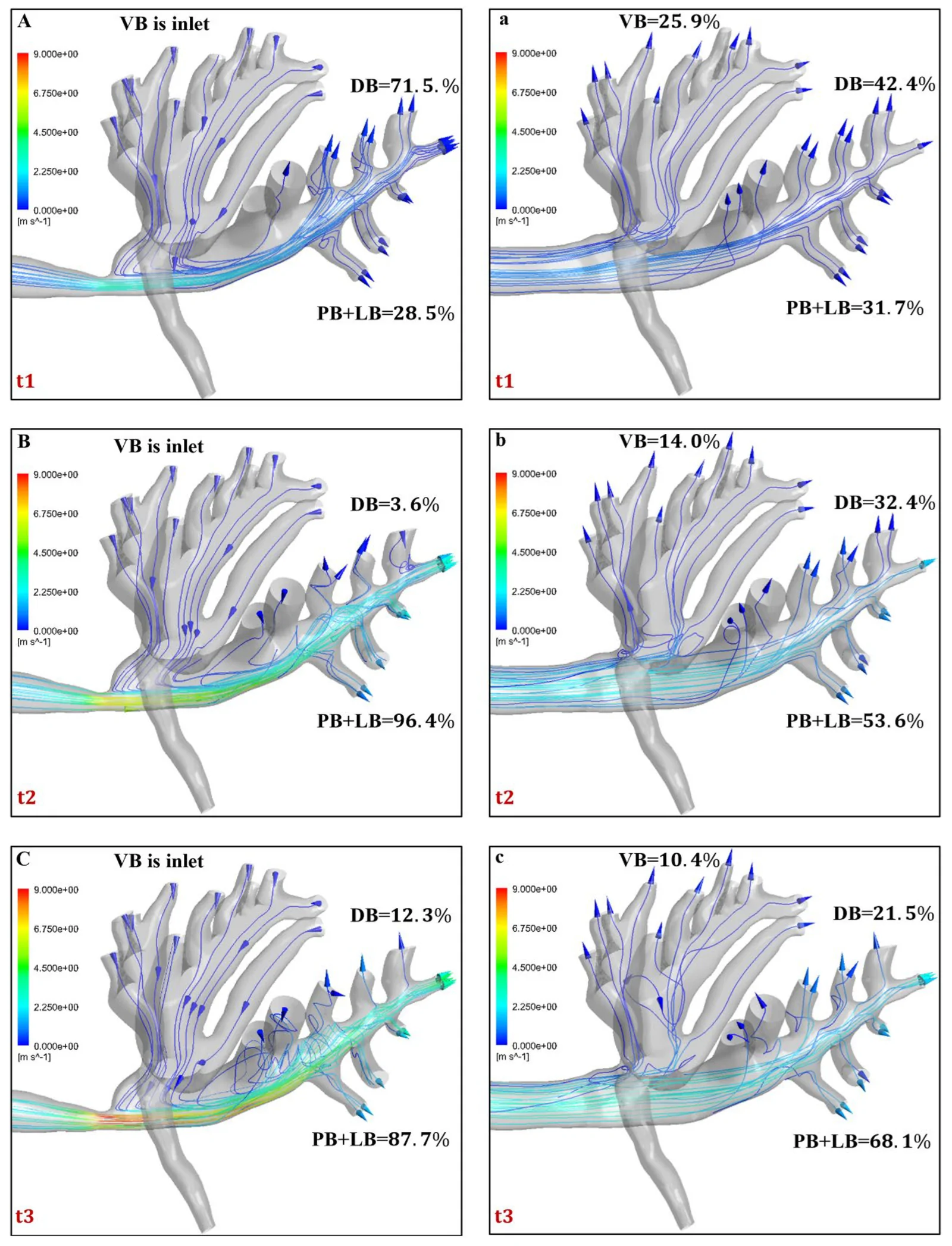
Simulations of airflow distribution in the lower respiratory tract and the proportion entering different regions during the dynamic inspiration process. Streamlines with arrows indicate the flow direct in each bronchus. Images labeled with capital letters (**A**–**C**) represent the original CT scan geometry with the ‘segmentum accelerans’, whereas those labeled with lowercase letters (**a**–**c**) show the modified geometry without the ‘segmentum accelerans’. Note: although the geometric configuration of the right bronchus is not identical to that of the left, the results obtained are comparable and yield the same conclusions.

Figure 9 demonstrates the airflow distribution during inhalation. Images labeled with capital letters A–C depict the results from original CT-scanned geometry, which includes the ‘segmentum accelerans’, whereas those labeled with lowercase letters (a–c) show the predictions from modified geometry without the narrowing structure. At the beginning of the inspiration process ($t_{1}$), the velocity within the trachea is low but is accelerated due to the presence of the ‘segmentum accelerans’ as the air approaches the lung. The negative pressure generated by this high-velocity airflow creates a suction effect (Bernoulli’s Principle) on the cranial sacs, causing a small amount of air to be drawn into the primary bronchus via the VB, as indicated by the streamline arrows in Figure 9A. During this low flowrate phase of inspiration, the air moves towards the bronchial outlets in various directions. Flowrate calculations indicates that approximately 71.5% of inhaled air is directed toward the DB outlets, while the remaining 28.5% flows towards the caudal sacs (via the LB and PB).

As inhalation continues and the ‘segmentum accelerans’ structure exerts an accelerating effect, the velocity of the air within the airway increases dramatically. By $t_{2}$, a jet-like flow is established at the first bifurcation of the intrapulmonary primary bronchus. This enables the majority of the airflow, which has strong convective inertia, to bypass the subsequent branch-offs and flow directly into the abdominal sacs linked to the primary bronchus. This pathway also presented relatively low flow resistance under the simulated conditions. Consequently, only a limited fraction of airflow is directed toward the DB outlets and caudal thoracic sacs (via the LB). As shown in Figure 9B, the caudal sacs as a whole receive 96.4% of the fresh air, with merely 3.6% being directed toward the DB outlets. At time point $t_{3}$ (see Figure 9C), as the inhalation nears its maximum, the intrapulmonary airflow distribution resembles that observed at *t*_2_. The high-velocity jet-like flow directs the majority of fresh air (87.7%) into the caudal sacs. Concurrently, the increased flow volume toward the DB outlets is likely associated with the greater momentum of the accelerated airflow through the ‘segmentum accelerans’, which enhances inertial effects and alters flow partitioning at the downstream bronchial bifurcations.

With regard to the simulation of airways without the ‘segmentum accelerans’ under the same boundary conditions, the airflow direction within the VB reverses. As the inhalation velocity progressively increases from $t_{1}$ to $t_{3}$, the airflow increasingly bypasses the VB, leading to a decrease in the proportion of air exiting the VB to the cranial sacs from 25.9% to 10.4% (see Figure 9a–c). Meanwhile, a distinct trend is observed where the air flowing out from the DB decreases with the increase in inhalation velocity. Additionally, an increasing proportion of fresh air flows into the caudal sacs, rising from 31.7% at $t_{1}$ to 68.1% at $t_{3}$. A reasonable hypothesis could be drawn that with the inhalation velocity further increased, the proportion of fresh air entering the caudal sacs will continue to rise, approaching conditions similar to those of the original airway geometry.

### 3.2. Expiratory Phase

**Figure 10:**
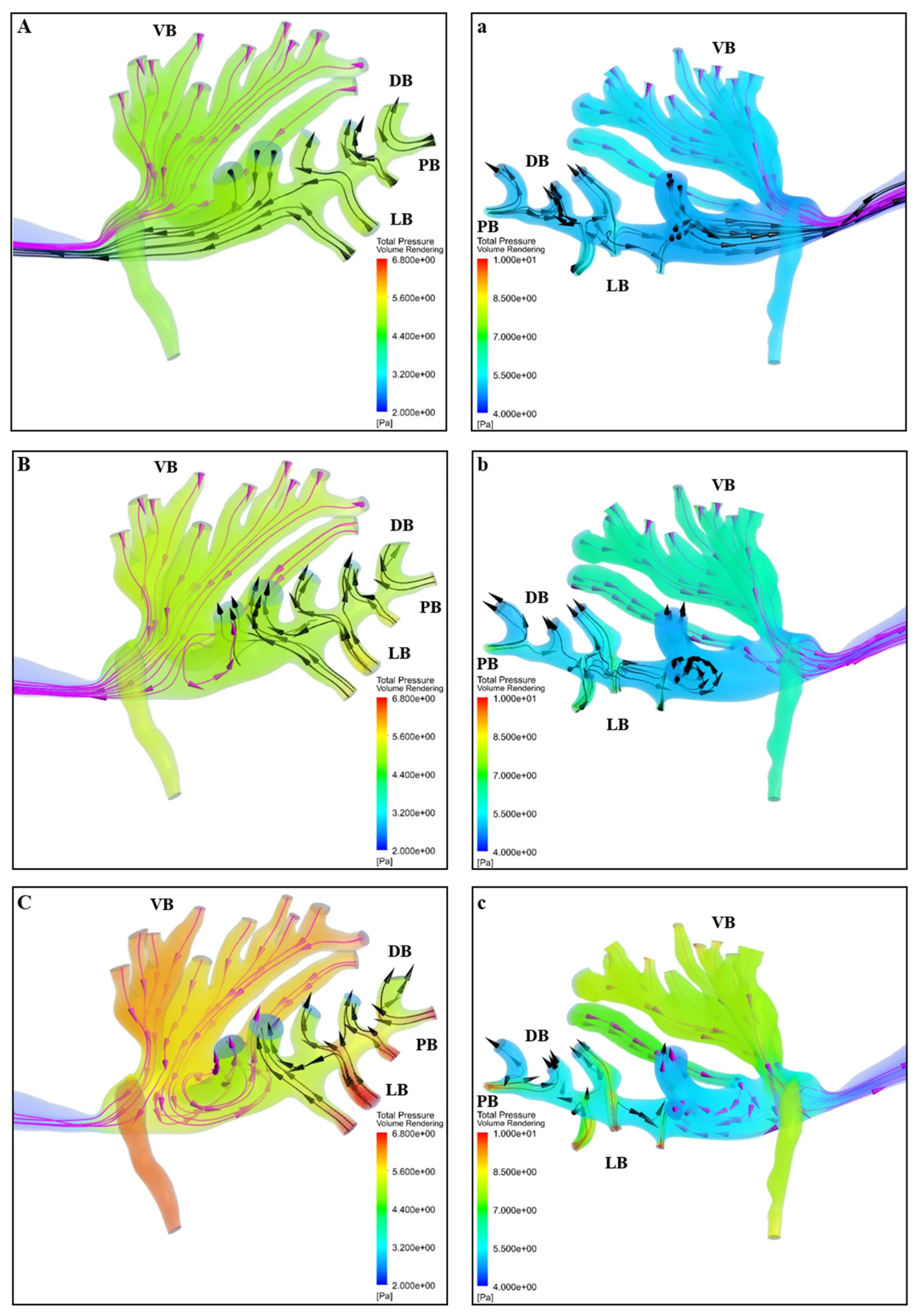
Flow characteristics in CT-scanned geometry during the exhalation phase under a DB pressure of 5 Pa with low (**A**,**a**), suitable (**B**,**b**), and high (**C**,**c**) pressure values for LB and PB. The capital letters (**A**–**C**) correspond to the left bronchus, whereas the lowercase letters (**a**–**c**) correspond to the right bronchus. The background volume-rendering contour illustrates the total pressure distribution. The black arrowed streamlines depict the direction of high-pressure airflow from the caudal sacs (via LB and PB), while the pink arrowed streamlines indicate the airflow direction from the cranial sacs (via VB).

Figure 10 illustrates the simulation results of the CT-scanned respiratory geometry with the DB at 5 Pa and varying pressures in the LB and PB—low, suitable, and high. The background volume-rendering contours indicate the total pressure distribution within both the left (capital letters) and right (lowercase letters) lower respiratory tracts. Black arrowed streamlines represent the direction of airflow originating from the caudal sacs (via the LB and PB), while pink arrowed streamlines represent the airflow direction from the cranial sacs (via the VB). When pressures in the LB and PB are relatively low (yet still exceeding the DB pressure), caudal-sac-derived airflow is distributed between the DB outlets and the tracheal pathway (see Figure 10A,a). At this stage, the air flowrate and pressure from the cranial sacs (via the VB) are also low, with airflow directed into the upper respiratory tract. With further contraction of the related muscles, air sac pressure rises, leading to elevated pressure at the LB and PB, along with increases in the mass flowrate and pressure at the VB. Upon reaching a critical pressure threshold (see Figure 10B,b), the predefined expiratory aerodynamic-valving condition is achieved. At this point, airflow from the VB is directed toward the upper respiratory tract and effectively suppresses retrograde caudal-sac airflow through the primary bronchus toward the trachea. Consequently, caudal-sac-derived airflow is preferentially redirected toward the DB outlets, which represent the entrance to the unresolved paleopulmonic pathway. As the imposed boundary pressures continue to increase, the pressure differences between the DB and the LB, PB, and VB boundaries become larger. Under these conditions, nearly all caudal-sac-derived airflow is redirected toward the DB outlets, and part of the cranial-sac boundary flow can also be redirected toward the DB outlets because of the increased pressure differences (see Figure 10C,c).

**Figure 11:**
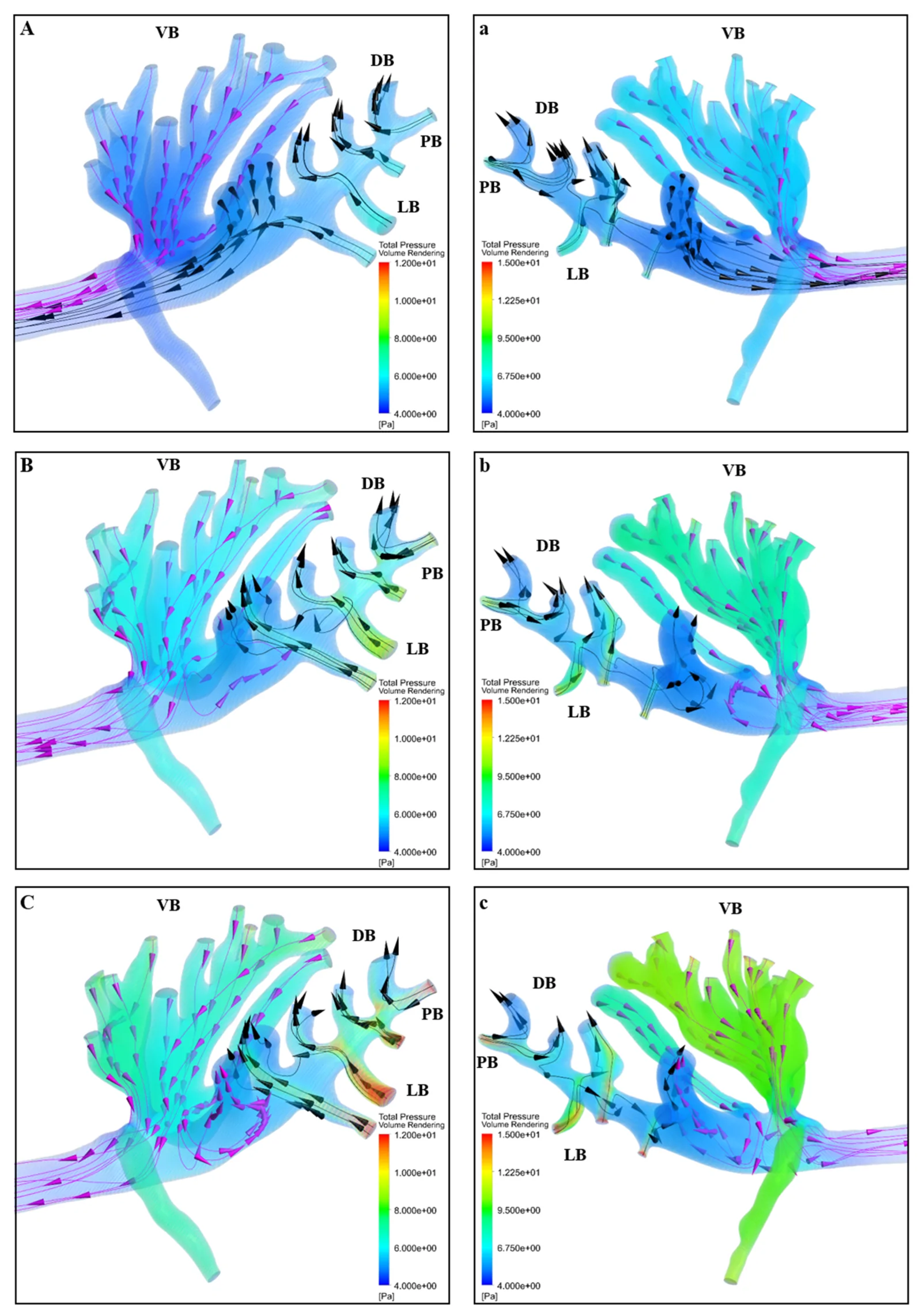
Flow characteristics in modified geometry during exhalation phase under a DB pressure of 5 Pa with low (**A**,**a**), suitable (**B**,**b**), and high (**C**,**c**) pressure values for LB and PB. The capital letters (**A**–**C**) correspond to the left bronchus, whereas the lowercase letters (**a**–**c**) correspond to the right bronchus. The background volume-rendering contour illustrates the total pressure distribution. The black arrowed streamlines depict the direction of high-pressure airflow from the caudal sacs (via LB and PB), while the pink arrowed streamlines indicate the airflow direction from the cranial sacs (via VB).

Regarding the effect of the ‘segmentum accelerans’ on airflow during exhalation, the local airway constriction increases flow losses and modifies airflow partitioning within the major bronchial system. This effect would theoretically facilitate the redirection of caudal-sac-derived airflow toward the DB outlets. Nevertheless, the subsequent simulations demonstrate that the same expiratory aerodynamic-valving pattern can still be reproduced in the absence of the ‘segmentum accelerans’, although higher caudal-sac boundary pressures (LB and PB) are required. Figure 11 shows the flow distribution results under the modified geometry at the same DB pressure of 5 Pa. The fresh air originating from the caudal sacs flows towards both the DB outlets and the tracheal pathway when the air sac pressure is low (see Figure 11A,a), with the distribution proportions determined by the pressure at the LB and PB. As the mass flowrate and pressure associated with the cranial-sac boundary flow increase in accordance with those of the caudal-sac boundaries, retrograde caudal-sac airflow toward the upper airway is almost completely suppressed, even in the absence of the ‘segmentum accelerans’ (see Figure 11B,b). Under this condition, caudal-sac-derived airflow is redirected toward the DB outlets, reproducing the expiratory aerodynamic-valving pattern defined in the present model. The critical caudal- and cranial-sac boundary pressures required to achieve this aerodynamic-valving condition are approximately 57% and 65% higher, respectively, than those required in the original CT-scanned geometry. As the pressure within the air sacs continues to rise, part of the cranial-sac boundary flow begins to be redirected toward the DB outlets, similar to the behavior observed under the original geometry conditions, driven by the significant pressure difference (see Figure 11C,c). Finally, these results indicate that the ‘segmentum accelerans’ is not a necessary condition for reproducing expiratory aerodynamic valving and major-bronchial flow redirection in the present model.

**Figure 12:**
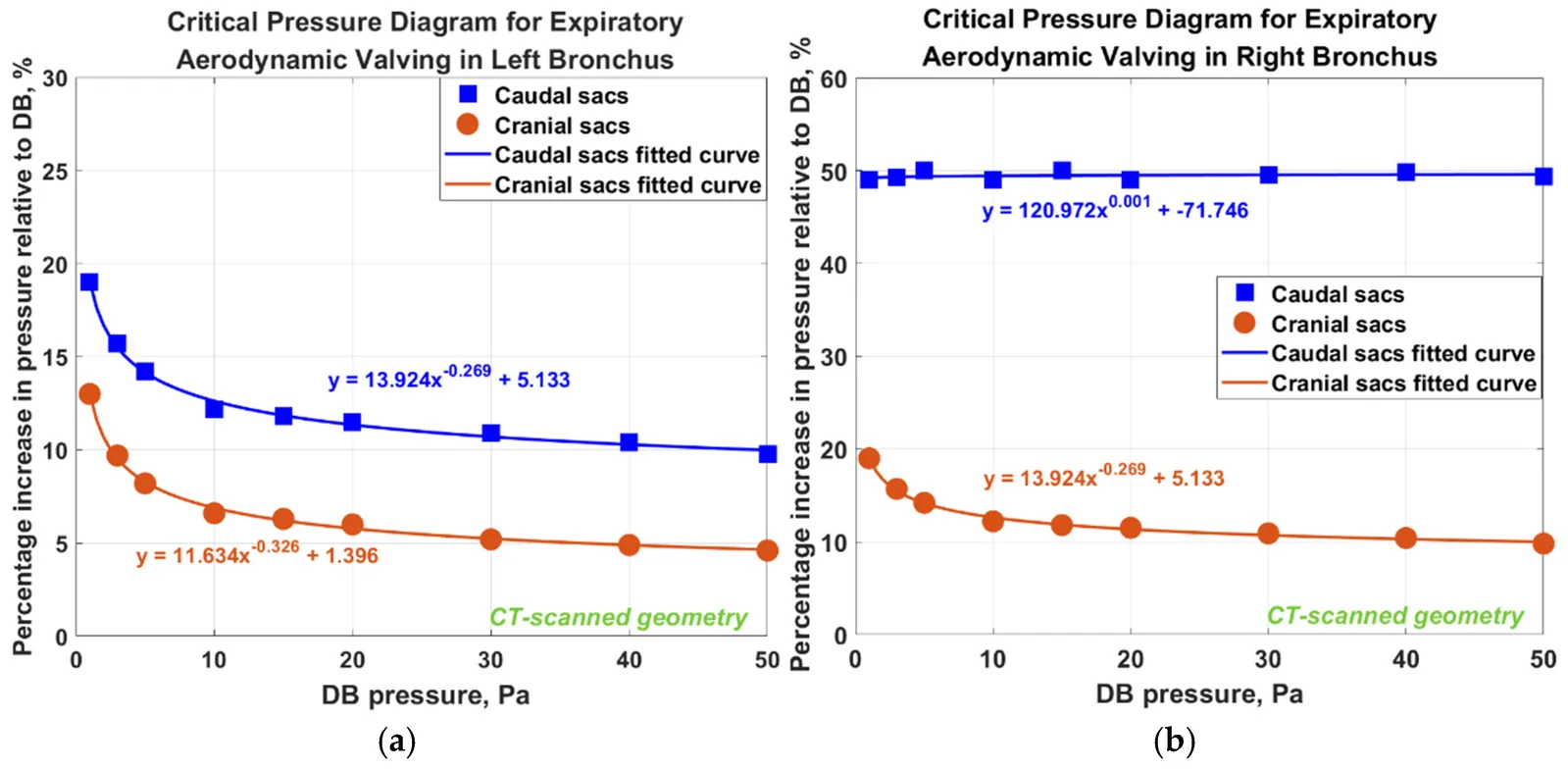
Critical pressures required for expiratory aerodynamic valving in (**a**) the left bronchus and (**b**) the right bronchus with original CT-scanned respiratory geometry.

In this study, the left and right bronchi exhibit inherent geometric differences, providing two distinct cases for analyzing airflow characteristics across different anatomical topologies. To quantitatively determine the critical pressure thresholds required to achieve the predefined expiratory aerodynamic-valving condition, we systematically examined numerous pressure combinations and constructed critical pressure maps for each bronchial geometry. Figure 12 demonstrates clearly that under the CT-scanned respiratory geometry, the critical pressure values and their trends differ between the left and right bronchi. At the onset of exhalation, when the DB boundary pressure is relatively small (DB = 1.0 Pa), the critical pressure required for the caudal sacs (LB and PB) in the left bronchus is approximately 19.0% higher than the DB pressure (see Figure 12a). This percentage decreases as the DB boundary pressure increases. When the DB boundary pressure reaches (DB = 50.0 Pa), the critical pressure needed for the caudal sacs (LB and PB) reduces to only about 9.8% above the DB. For the cranial sacs (VB), the overall critical pressure is lower than that for the caudal sacs, although the trend remains similar. In contrast, as shown in Figure 12b, the pressure required at the caudal sac in the right bronchus remains nearly constant at around 49.2% higher than the DB pressure, whereas the ratio of the critical pressure for the cranial sacs to the DB pressure gradually decreases with increasing DB boundary pressure. Overall, in the CT-scanned geometry containing the ‘segmentum accelerans’, achieving expiratory aerodynamic valving required an increase in air-sac boundary pressure relative to the DB boundary pressure. However, this percentage increase decreased as the DB boundary pressure increased.

**Figure 13:**
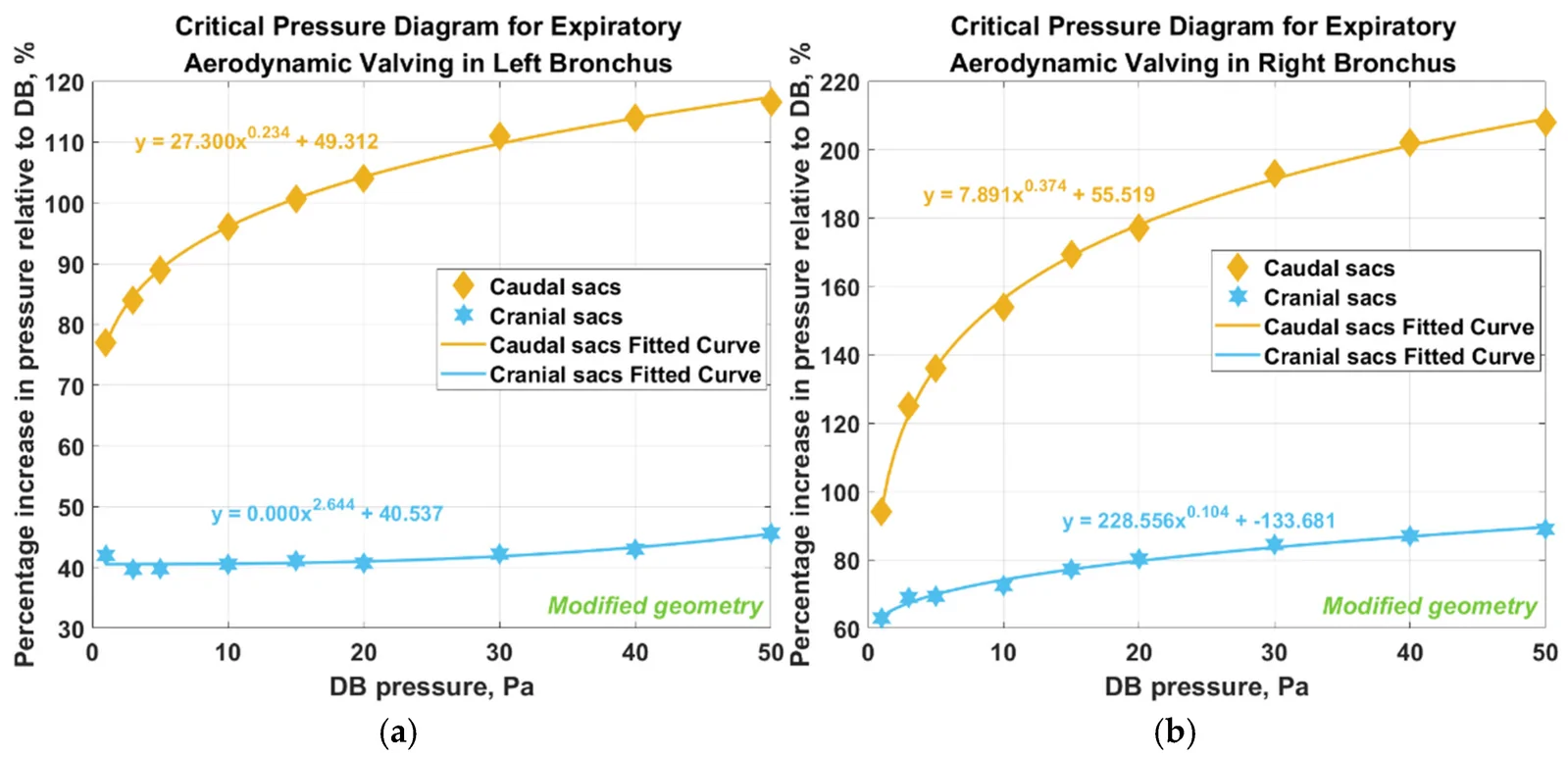
Critical pressures required for expiratory aerodynamic valving in (**a**) the left bronchus and (**b**) the right bronchus with modified respiratory geometry lacking the ‘segmentum accelerans’.

Under the modified respiratory geometry lacking the ‘segmentum accelerans’ structure, the overall trend of the ratio of critical pressure to DB pressure exhibits a gradual increase, which is in stark contrast to the results obtained with the CT-scanned geometry. For the left bronchus, the percentage increase relative to the DB pressure rises from about 77.0% at DB = 1.0 Pa to 116.0% at DB = 50.0 Pa for the caudal sacs (see Figure 13a). In comparison, the increase for the cranial sacs (VB) is relatively minor. Moreover, for the right bronchus, both the critical pressure required for the caudal sacs (LB and PB) and that for the cranial sacs (VB) increase significantly with the rise in the DB pressure (Figure 13b).

### 3.3. Fluid Density Effects

When fluid density decreases, marked shifts in respiratory airflow distribution become evident during inspiration, as outlined in Table 3. In the CT-derived geometry, a predominant fraction of fresh air (about 84.2%) is directed toward the DB outlets at the initial inspiratory stage of $t_{1}$, with only a limited proportion (15.8%) penetrating into the caudal air sacs. As inspiratory flow intensifies sequentially from $t_{1}$ through $t_{3}$, the proportion of airflow towards to the DB outlets decreases progressively to 68.8%, though the preferential caudal-directed inspiratory valving pattern is not established. Notably, no fresh air enters the cranial sacs via the VB during the entire inhalation process. Instead, a small amount of air flowed from the cranial sacs toward the PB, consistent with the situation before the density change.

Furthermore, the convective inertia of fresh air flowing into the intrapulmonary bronchi from the PB is further reduced when the ‘segmentum accelerans’ is removed in the modified geometry. Consequently, at time point $t_{1}$, nearly 45.2% of the inspired air is directed into the cranial air sacs via the ventral bronchi (VB), with only approximately 4.5% reaching the caudal sacs. As the convective inertial force increases from $t_{1}$ to $t_{3}$, although the proportion of air flowing into the cranial sacs has decreased from 45.2% to 17.8%, the air still lacks sufficient momentum to enter the caudal sacs (accounting for only 19.5% at $t_{3}$). Instead, the majority of air (62.7% at $t_{3}$) is directed toward the DB outlets, thus failing to reproduce the preferential caudal-directed inspiratory valving pattern observed under sea-level density.

**Figure 14:**
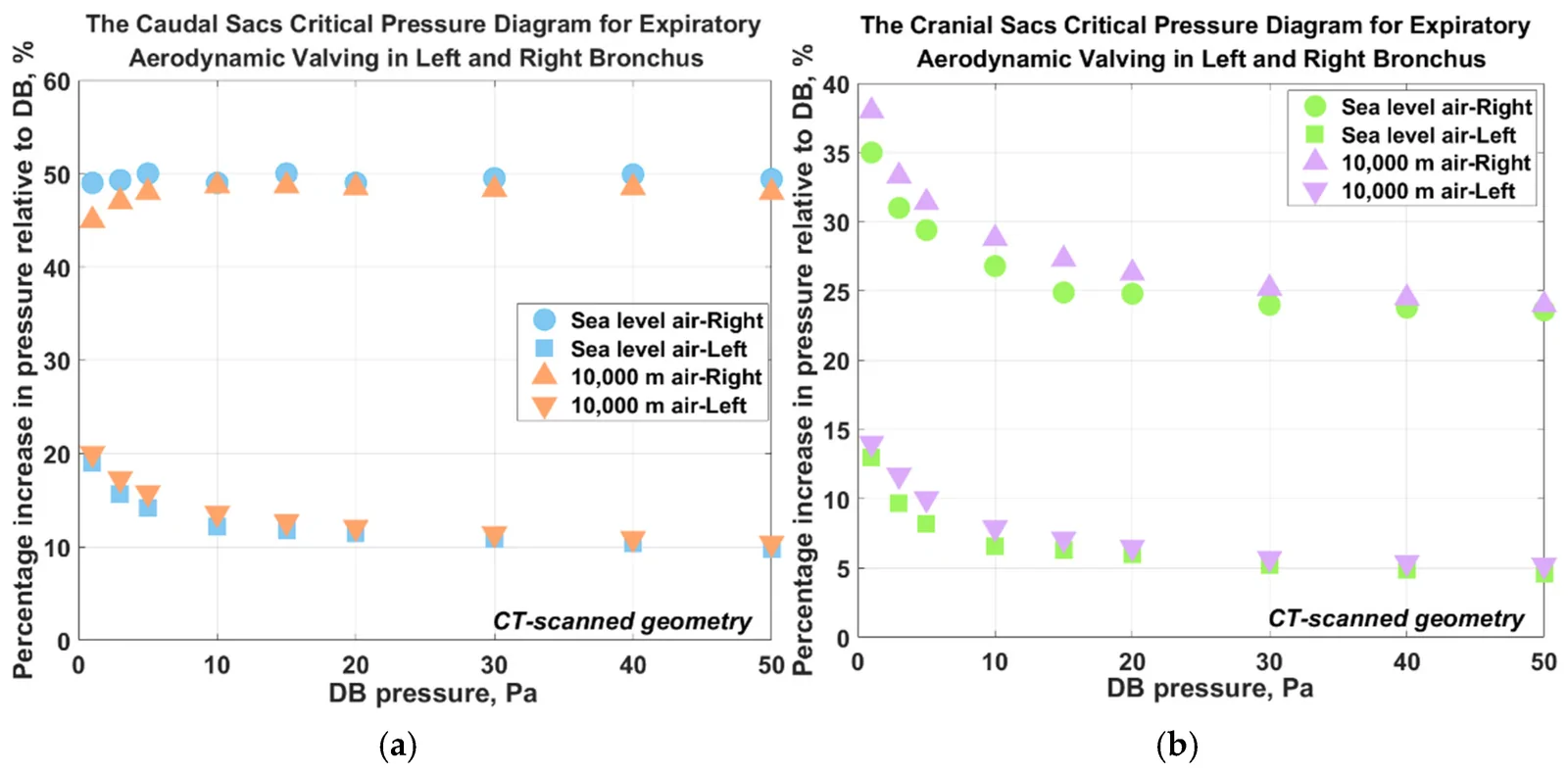
The effects of air density on the critical air sac pressures required for establishing expiratory unidirectional flow: (**a**) for caudal sacs, (**b**) for cranial sacs.

Regarding the expiratory process, Figure 14 presents the critical air-sac boundary pressures required to achieve the predefined expiratory aerodynamic-valving condition following changes in air density. For the caudal sacs (Figure 14a), reducing air density has differential effects on the critical pressures of the left and right bronchi: the critical pressure for the right bronchus decreased, while that for the left bronchus increased. Conversely, for the cranial sacs (Figure 14b), decreasing air density results in increased critical pressures on both sides. Overall, at lower DB pressures, changes in air density cause more significant variations in the critical pressures, and this effect diminishes as the prescribed DB boundary pressure increases.

## 4. Discussion

### 4.1. Fluid Mechanics of Aerodynamic Valving and Major-Bronchial Airflow Routing

Airflow distribution within the avian respiratory system is governed by the combined effects of airway geometry, pressure gradients, and fluid inertia. Pressure gradients provide the driving force for airflow, whereas inertia and local airway geometry influence flow direction and partitioning at bronchial bends and bifurcations. These interacting factors determine preferential airflow pathways within the major bronchial system and provide the physical basis for aerodynamic valving associated with physiological unidirectional pulmonary airflow.

**The inspiratory phase:** The anatomical structure of avian lungs revealed that the primary bronchus (PB) connected directly to the caudal sacs, particularly the abdominal sacs. During the initial phase of inspiration (see Figure 9A), as no single factor dominates the flow, fresh air was distributed between the caudal-sac pathways and the DB outlets.

As the negative pressure within the air sacs intensified, both flow velocity and rate increased. For fresh air entering the intrapulmonary bronchi from the PB, the pressure gradient, convective inertial force, and path of least resistance all directed airflow toward the abdominal sacs, with only a small fraction was directed toward the DB outlets (see Figure 9B). The simulation results from the present study aligned with those of Banzett et al. [21], indicating that aerodynamic valves did not operate at full efficiency and were influenced by flow velocity. Additionally, Powell and Hempleman’s research [27], which observed higher-than-expected CO_2_ partial pressures in the caudal thoracic sacs, suggested that inspiratory airflow in the avian lung may be more complex than the idealized pattern in which newly inspired air is routed exclusively toward the caudal air sacs. This interpretation is broadly consistent with the present simulations, which showed that a fraction of the inspired airflow was directed toward the DB outlets rather than exclusively toward the caudal-sac boundaries.

Previous studies [16,28] also emphasized the critical role of fluid inertia, more specifically the convective inertia, in forming the inspiratory aerodynamic valving. Our findings corroborated this view, but further indicated that the specific geometric configuration of the intrapulmonary primary bronchus and its connection to the caudal sacs plays a central role in establishing inspiratory aerodynamic valving. Additionally, our results demonstrated that the presence of the ‘segmentum accelerans’ structure mainly enhanced the convective inertia of the fluid and impacted the efficiency of inspiratory valve formation, which aligned with the views of Maina and Africa [15]. Importantly, this narrowing was not itself the inspiratory valve and was not required to reproduce the inspiratory aerodynamic-valving pattern.

In conclusion, inspiratory aerodynamic valving is achieved through the coupled effects of the specific bronchial geometric topology and the negative pressure generated by the caudal sacs. These findings provide a possible fluid–mechanical explanation for how inspiratory aerodynamic valving may occur in species lacking a ‘segmentum accelerans’ [1,29], although direct interspecific extrapolation requires further comparative investigation.

**The expiratory phase:** Similarly, at the beginning of expiration, fluid driven by pressure gradients exited from both the caudal and cranial sacs. Fresh air from the abdominal sacs (via the LB and PB) and caudal thoracic sacs (via the LB) entered the bronchi and merged within the airway. At this point, two paths were available for the merged flow: one leading toward the DB outlets and the other returning toward the upper respiratory tract through the primary bronchus. It was demonstrated in the present study that air from the caudal sacs indeed followed both of the two routes (see Figure 10A) at the initial exhalation phase. This finding was consistent with Kuethe’s research [17], which emphasized that flow velocity and airway inclination significantly influenced the direction of airflow.

As the pressure in the caudal and cranial sacs continued to increase, airstream from the cranial sacs (via the VB) progressively suppressed the pathway for fresh air from the caudal sacs to return to the upper respiratory tract via the way it came. This major-bronchial flow-redirection pattern was reproduced in both the original and modified geometries (see Figure 10 and Figure 11), indicating that it was not dependent on the presence of the ‘segmentum accelerans’. At this stage, the airstream via the VB acted as a strong momentum barrier. Consequently, air from the caudal sacs (via the LB and PB) would have to expend substantial energy to overcome this barrier before entering the upper respiratory tract, resulting in significant energy loss. Therefore, in accordance with the principle of least resistance, the fresh air from caudal sac tended to follow the path of least resistance, flowing toward the DB outlets. This was the key mechanism underlying the establishment of the expiratory valve.

With further increase in pressures in the air sacs, the airstream from the cranial sacs (via the VB) formed a more effective momentum barrier. Simultaneously, the fresh air from the caudal sacs (via the LB), driven by a strong pressure gradient between the DB and the LB, and aided by sufficient convective inertia, was directed straightly toward the DB outlets (see Figure 10B). From an alternative perspective, the caudal-sac-derived airstream directed toward the DB outlets also established a momentum barrier against the airstream from the VB, which prevented most of the air from the VB from flowing back into the caudal sacs or the DB outlets. Consequently, the two airstreams interacted and mutually restricted each other.

It should be noted that, according to the physiological and anatomical structure of most avian species (e.g., domestic chicken), the caudal sacs were characteristically surrounded by more robust muscle groups when compared to the cranial ones [7]. As a result, the pressure generated by the caudal sacs generally exceeded that of the cranial sacs. This finding aligned with our study, which demonstrated that, during the establishment of expiratory unidirectional airflow, the critical pressure required for the cranial sacs was always lower than that of the caudal air sacs (see Figure 12). Because the expiratory analysis was performed under steady-state conditions, temporal phase differences between the caudal- and cranial-air-sac pressures were not represented. Previous work has shown that air-sac compliance can influence airflow pathways [30], and these time-dependent effects should therefore be examined in future transient models. This suggested that air-sac compliance significantly influenced airflow pathways.

In conclusion, the expiratory simulations indicate that airflow routing within the major bronchial system is governed by the interaction of imposed pressure gradients, convective inertia, and local flow losses. As the cranial-sac boundary flow increased, retrograde caudal-sac airflow through the primary bronchus was progressively suppressed, and caudal-sac-derived airflow was redirected toward the DB outlets. Thus, the present study explained the aerodynamic-valving mechanism at the level of the major bronchi. Finally, the ‘segmentum accelerans’ facilitated expiratory aerodynamic valving by promoting major-bronchial flow redirection and reducing the air-sac boundary pressures required to reach the predefined expiratory valving condition.

### 4.2. The Effect of Fluid Density

From a theoretical standpoint, changes in fluid density within the airway primarily affected two flow parameters [31]. The first was the flow loss coefficient, which included both the frictional and local loss coefficients and was related to the Reynolds number. The second was the convective inertia force. In this study, when the density was reduced to approximately one-third of its original value, the inertial forces decreased markedly. During inhalation, this reduction caused the fresh air to flow along the PB toward the lungs via the DB outlets. Notably, under specific bronchial topologies, this airflow could even extend to the cranial sacs. This observation aligned with the experimental findings of Banzett et al. [21], whose study demonstrated that the inspiratory valve function in the goose lung depends on both the velocity and density of the gas. Specifically, when the gas density was reduced (for example, by using helium) or the gas flowrate was lowered, the normal inspiratory valve mechanism failed, allowing the tracer gas to enter the cranial sacs [21].

During exhalation, the substantial reduction in density appeared to have a limited effect on the performance of the expiratory aerodynamic valve. As shown in Figure 14, across all the investigated pressure values at the DB boundary, the variation in the required critical air sac pressure remained within 5%. This was likely because the momentum barrier created by the airstream from the cranial sacs, along with the interaction between the two airstreams (cranial and caudal), are the dominant factors in forming the expiratory valve. In contrast, the formation of the inspiratory valve was primarily governed by convective inertial forces.

These results indicate that inspiratory aerodynamic valving was more sensitive to changes in fluid density and convective inertia than expiratory valving. In contrast, expiratory valving in the present model depended more strongly on the imposed pressure conditions and the interaction between the cranial- and caudal-sac boundary airstreams.

### 4.3. Limitations

The major limitation arises from the spatial resolution of the CT-based reconstruction. The small paleopulmonic parabronchi connecting the DB and VB could not be reliably segmented, although these anatomical connections are well established [5]. Consequently, the present model does not directly simulate the characteristic unidirectional airflow within the paleopulmonic parabronchi. The present results should thus be interpreted as describing aerodynamic valving and airflow routing within the major bronchial system, rather than as a complete simulation of intrapulmonary airflow.

For the expiratory simulations, all segmented terminal VB branches were treated as surrogate cranial-air-sac boundaries. This simplification does not fully reproduce the physiological heterogeneity of VB connections and may affect local pressure distributions, airflow partitioning, and the magnitude and spatial distribution of the modeled cranial-sac airstream. In addition, the steady-state pressure-boundary approach does not reproduce time-dependent air-sac compliance or possible phase differences among air-sac groups. Therefore, the critical pressures reported here should be regarded as model-specific thresholds for major-bronchial expiratory aerodynamic valving rather than direct physiological thresholds for whole-lung unidirectional airflow. Future studies incorporating higher-resolution imaging, explicit reconstruction of the parabronchial network, and physiologically based transient air-sac boundary conditions will be required to evaluate these effects.

A further anatomical limitation concerns the proximal ventrobronchial region. Owing to the spatial resolution of the clinical CT dataset (slice thickness, 0.5 mm), the thin septa separating adjacent ventrobronchial ostia could not be reliably resolved. Consequently, several proximal ventrobronchial branches and their ostia could not be represented as anatomically distinct structures, and the large cranially projecting first ventrobronchus (V1) was not represented as a distinct airway in the final CFD geometry. The present model therefore does not reproduce the complete native ventrobronchial anatomy of the chicken. During inspiration, because V1 is oriented cranially, opposite to the dominant caudal direction of the inspiratory core flow, direct entry of high-momentum inspired airflow into this branch would be mechanically unfavorable, particularly as inspiratory velocity increases. During expiration, V1 acts together with the other ventrobronchi to contribute to the cranial-side airstream entering the primary bronchus, which interacts with caudal-sac-derived airflow and contributes to the momentum barrier associated with expiratory aerodynamic valving. Omission of V1 may therefore alter the local magnitude and spatial distribution of this expiratory airstream, while the remaining ventrobronchial pathways can still contribute to the same flow-redirection mechanism. We therefore do not expect the absence of V1 to determine whether the major inspiratory or expiratory flow-rectification patterns can occur in the present model, although its omission and the simplified representation of the proximal ventrobronchial ostia may quantitatively affect local flow separation, pressure distribution, airflow partitioning, and aerodynamic-valving efficiency. Higher-resolution imaging, particularly micro-CT, will be required to quantify these local effects and the specific contributions of V1 and the individual ventrobronchial ostia.

Moreover, Brown et al. [18] hypothesized that the pressure differential between the air sacs and the bronchial lumen tends to cause bronchial collapse, forcing air from the caudal sacs into the paleopulmonic parabronchi. Conversely, air from the cranial sacs could not flow caudally through the primary bronchus and could only move cranially to exit the lung via the trachea. In our study, since the geometry of the simulated respiratory tract remained unchanged, we could not determine whether airway collapse contributed to expiratory aerodynamic valving. Additionally, this phenomenon had not been confirmed by any other study. Nevertheless, the present model reproduced major-bronchial expiratory flow redirection without incorporating airway collapse, suggesting that the interaction between the cranial- and caudal-sac airstreams may contribute to expiratory aerodynamic valving.

Furthermore, the bidirectional airflow within the neopulmo, which typically occupied the lateral regions of the lungs, was not considered in this study. Future research can focus on investigating the airflow characteristics within the neopulmo and its dynamic distribution between the palaeopulmo and the neopulmo during the respiratory cycle. Finally, additional studies are warranted to investigate the topological structure of the respiratory tract of birds inhabiting different environments, particularly across various altitudes. These investigations could provide further insights into the relationship between natural selection and the development of unidirectional airflow mechanisms within the lungs.

Finally, the present study was restricted to the respiratory system of the domestic chicken and therefore does not address the broader diversity of mechanisms underlying unidirectional pulmonary airflow among sauropsids. Previous studies have demonstrated unidirectional pulmonary airflow in the American alligator, savannah monitor lizard, and green iguana despite substantial differences in pulmonary architecture [4,32,33], and these findings have been reviewed in a broader comparative and evolutionary context by Cieri and Farmer [34]. Comparative studies indicate that neither the characteristic avian air-sac system nor a typical avian bronchial arrangement is an absolute prerequisite for the generation of unidirectional airflow. To date, an avian-type ‘segmentum accelerans’ has not been documented in these non-avian taxa, although targeted comparative investigations of this structure remain limited. Notably, localized bronchial constrictions associated with jetting and aerodynamic valving have been described in the savannah monitor lizard, but these structures have not been identified as a ‘segmentum accelerans’. Future comparative anatomical and CFD studies across avian and non-avian sauropsids may therefore help clarify whether the ‘segmentum accelerans’ represents an avian specialization or whether functionally analogous acceleratory regions occur more broadly.

## 5. Conclusions

To provide new insights into the fluid mechanics of avian aerodynamic valving, a respiratory tract model of a domestic chicken was reconstructed based on CT-scanned data. The validated numerical model was analyzed using CFD-assisted simulations to systematically and comprehensively characterize the airflow dynamics within the airway under various conditions. The qualitative and quantitative results from the present study revealed that:

- During inspiration, the interaction between bronchial geometry, pressure gradients, and convective inertia preferentially routed inspired airflow toward the caudal-sac pathways.
- During expiration, the cranial-sac airstream generated a momentum barrier that progressively suppressed retrograde caudal-sac airflow through the primary bronchus and redirected it toward the DB outlets and, physiologically, into the paleopulmonic pathway.
- At the level of the major bronchial system, aerodynamic valving resulted from the coupled effects of bronchial topology and air-sac pressure conditions rather than from a discrete anatomical valve.
- The ‘segmentum accelerans’ facilitated aerodynamic valving during both inspiration and expiration, but was not essential for major-bronchial flow rectification; its removal elevated the air-sac boundary pressures required to achieve both expiratory and inspiratory aerodynamic valving.
- Fluid density influenced aerodynamic valving primarily by altering convective inertia, with a substantially greater effect during inspiration than on the critical pressure conditions for expiratory valving.

## Figures and Tables

**Table 1:** Summarization of the boundary conditions applied in this study.

Boundaries	Inhalation	Exhalation
Nose, N1	Velocity inlet	Pressure outlet, 0 Pa
Mouth, N2	Wall	Pressure outlet, 0 Pa
VB	Pressure outlet, 0 Pa	Pressure inlet
LB and PB	Pressure outlet, 0 Pa	Pressure inlet
DB	Pressure outlet, 0 Pa	Pressure inlet, P=1,3,5,10,15,20,30,40,50 Pa
Entire airway surfaces	Wall	Wall
Air density	$\rho =1.225\;kg/m^{3}$	$\rho =1.225\;kg/m^{3};\rho =0.413\;kg/m^{3}$
Air viscosity	μ=1.789 kg/(m·s)	μ=1.789 kg/(m·s);μ=1.420 kg/(m·s)

Note: All pressures in this study are relative to atmospheric pressure.

**Table 2:** Detailed information about the experimental apparatus.

Name	Type/Manufacturer	Unit	Resolution
3D-printed 1:1 respiratory tract model	Acrylonitrile Butadiene Styrene (ABS)	-	-
Air pump	Quick Take 30, SKC, USA	L/min	0.01
Flow meter	4000 Series, TSI, USA	L/min	0.01
Pressure meter	Testo 510, Germany	Pa	1.0

**Table 3:** Effects of air density on the percentage of airflow exiting each bronchus outlet during inhalation.

Air Density	Time	CT-Scanned Geometry			Modified Geometry		
At sea level		DB	LB&PB	VB	DB	LB&PB	VB
	$t_{1}$	71.5%	28.5%	/	42.4%	31.7%	25.9%
	$t_{2}$	3.6%	96.4%	/	32.4%	53.6%	14.0%
	$t_{3}$	12.3%	87.7%	/	21.5%	68.1%	10.4%
At 10,000 m		DB	LB&PB	VB	DB	LB&PB	VB
	$t_{1}$	84.2%	15.8%	/	50.3%	4.5%	45.2%
	$t_{2}$	71.9%	28.1%	/	61.1%	15.7%	23.2%
	$t_{3}$	68.8%	31.2%	/	62.7%	19.5%	17.8%

## Data Availability

Data will be made available upon request.
